# Impact of extended-course oral nirmatrelvir/ritonavir (Paxlovid) in established Long COVID: Case series and research considerations

**DOI:** 10.21203/rs.3.rs-3359429/v1

**Published:** 2023-09-20

**Authors:** Alison K. Cohen, Toni Wall Jaudon, Eric M. Schurman, Lisa Kava, Julia Moore Vogel, Julia Haas-Godsil, Daniel Lewis, Samantha Crausman, Kate Leslie, Siobhan Christine Bligh, Gillian Lizars, JD Davids, Saniya Sran, Michael J. Peluso, Lisa McCorkell

**Affiliations:** University of California San Francisco & Patient-Led Research Collaborative; Patient-Led Research Collaborative & University of Arkansas for Medical Sciences; Patient-Led Research Collaborative; Scripps Research Translational Institute and Patient-Led Research Collaborative; Patient-Led Research Collaborative; University of California San Francisco; Patient-Led Research Collaborative

**Keywords:** Long COVID, nirmatrelvir/ritonavir (Paxlovid), case series, post-acute sequelae of SARS-CoV-2 (PASC)

## Abstract

**Background:**

Prior case series suggest that a 5-day course of oral Paxlovid (nirmatrelvir/ritonavir) benefits some people with Long COVID, within and/or outside of the context of an acute reinfection. To the best of our knowledge, there have been no prior case series of people with Long COVID who have attempted longer courses of nirmatrelvir/ritonavir.

**Methods:**

We documented a case series of 13 individuals with Long COVID who initiated extended courses (>5 days; range: 7.5–30 days) of oral nirmatrelvir/ritonavir outside (n=11) of and within (n=2) the context of an acute SARS-CoV-2 infection. Participants reported on symptoms and health experiences before, during, and after their use of nirmatrelvir/ritonavir.

**Results:**

Among those who took a long course of nirmatrelvir/ritonavir outside of the context of an acute infection, some experienced a meaningful reduction in symptoms, although not all benefits persisted; others experienced no effect on symptoms. One participant reported intense stomach pain that precluded her from continuing her course. Among the two participants who took a long course of nirmatrelvir/ritonavir within the context of an acute reinfection, both eventually returned to their pre-re-infection baseline.

**Discussion:**

Long courses of nirmatrelvir/ritonavir may have meaningful benefits for some people with Long COVID but not others. We encourage researchers to study who, how, and why nirmatrelvir/ritonavir benefits some and what course length is most effective, with the goal of informing clinical recommendations for using nirmatrelvir/ritonavir and/or other antivirals as a potential treatment for Long COVID.

## Introduction

Long COVID is an often debilitating, multisystemic illness that occurs following a SARS-CoV-2 infection^1^ that affects ≥ 76 million people worldwide.^2,3,4^ It can last for months, years, or potentially a lifetime. Over 1% of all American adults currently have severe activity limitations due to Long COVID.^5^ Long COVID symptoms can include brain fog, fatigue, post-exertional malaise (PEM), autonomic dysfunction, headache, tinnitus, blurred vision, loss or alteration of smell or taste, palpitations, gastrointestinal symptoms, changes in sexual desire or capacity, menstrual cycle impacts, chronic cough, and chest pain.^6,7^

The pathophysiology underlying Long COVID is an area of major ongoing investigation. After acute COVID-19, some patients do not fully clear the SARS-CoV-2 virus, and viral reservoirs may persist over long periods of time.^8,9,10,11,12^ For example, SARS-CoV-2 viral RNA and protein can persist in blood and/or tissue beyond the acute phase of infection.^13^

The oral antiviral Paxlovid (nirmatrelvir/ritonavir), when administered for acute COVID-19, can reduce viral activity,^14^ symptom intensity, fatalities,^15^ and the likelihood of developing Long COVID.^16^ Prior case series^17,18^ suggested symptomatic improvement in people with Long COVID who completed a 5-day course of nirmatrelvir/ritonavir for symptom management or in response to a subsequent COVID-19 infection. For some cases, symptoms recurred following completion of treatment, suggesting that longer courses may be needed.

Here, we report 13 cases of people with Long COVID who took > 5-day courses of nirmatrelvir/ritonavir and who described their lived experience of the impact of this agent on their Long COVID illness trajectory. These cases emphasize the need to study longer courses of antiviral therapy for Long COVID and lend further support to the pursuit of clinical trials testing various durations of nirmatrelvir/ritonavir therapy in people experiencing Long COVID (e.g., NCT05668091, NCT05595369, NCT05576662).

## Methods

We compiled cases identified using two different methods. First, individuals who had previously shared aspects of their experience with nirmatrelvir/ritonavir in public forums were invited to participate. Second, others heard about the project via word-of-mouth and contacted us to share their experience. Participants chose to either share pre-existing written text (e.g.,, text shared in public forums, notes they wrote to clinicians) or talk with one of the authors about their experience. As needed, participants answered follow-up questions in writing. All participants consented to the presentation of their cases in this manuscript and had the opportunity to review and edit the written description of their experience.

We included individuals who reported a diagnosis of Long COVID and who reported taking > 5 days of nirmatrelvir/ritonavir at some point following their diagnosis. Participants typically reported on their experiences within 1 month of completing their course of nirmatrelvir/ritonavir, with some participants providing updates while on nirmatrelvir/ritonavir, and others drawing from contemporaneous notes and/or medical records documenting their experiences. Because participants were not asked to share any identifiable information, this study was self-certified as exempt from Institutional Review Board review.

## Results

### Case #1

In March 2020, a 56-year-old man with no prior medical history developed symptoms consistent with mild COVID-19. Confirmatory testing was not available at the time. Initially, most symptoms resolved, except for ongoing diarrhea. In August 2020, he developed symptoms of Long COVID, including severe fatigue, hypersomnia, brain fog, exercise intolerance, PEM, loss of hearing, photosensitivity, headaches lasting up to a week at a time, symptoms of vocal cord dysfunction, insomnia, elevated heart rate when standing and upon minor exertion, balance disturbance, vestibular dysfunction, and joint pain. He completed the primary SARS-CoV-2 vaccine series in February/March 2021 (Spikevax) and booster doses in November 2021 (Spikevax), May 2022 (Comirnaty), and November 2022 (Comirnaty).

In September 2022, he completed a five-day course of nirmatrelvir/ritonavir outside of the context of an acute COVID-19 infection, with no change in symptoms. In December 2022, he completed a 15-day course of nirmatrelvir/ritonavir outside of the course of an acute COVID-19 infection. Concomitant medications included cetirizine, famotidine, nattokinase, serrapeptase, and vitamins B, C, and D3. He began experiencing some improvement in symptoms during days 5–10, with more substantial improvement after 10 days of treatment. Following the 15-day course, he reported feeling substantially more energetic and that his muscles no longer felt like “dead weights.” He also reported improvement in his neurocognitive symptoms, including decreased photosensitivity, and improved clarity of thought, memory, and word-finding ability. One week after completing the course, he reported almost complete resolution of Long COVID symptoms. For example, he could walk continuously for 45 minutes and lightly jog 3–4 times for approximately 1 minute during exercise, with no exacerbation of symptoms or PEM. He also noted that arthritis in his fingers resolved after this course. Although his symptoms recurred to a certain extent within four weeks of completing the nirmatrelvir/ritonavir course, he reported an improved baseline.

### Case #2

In March 2022, a 45-year-old woman with no pertinent medical history developed mild COVID-19. The infection was confirmed by nucleic acid amplification testing. Prior to her infection, she was a physically active athlete and had received the primary two-shot series of COVID-19 vaccines (Spikevax) in January/February 2021, and a Spikevax booster in November 2021. About three weeks after her acute infection, she developed Long COVID symptoms, including orthostatic intolerance, tachycardia, short-term memory loss, word-finding difficulties, fatigue, PEM, and symptoms of mast cell activation syndrome (MCAS) including food intolerances, rashes, and headaches. She was diagnosed with MCAS and postural orthostatic tachycardia syndrome (POTS); she attained partial relief with beta blockers, although her blood pressure regularly fluctuated between 80–145 (systolic) and 60–80 (diastolic). When her blood pressure was low, she experienced weakness, shortness of breath, light-headedness, and episodes of fainting. Her symptoms of fatigue, orthostatic intolerance, and PEM worsened following a COVID-19 vaccine booster (Comirnaty) in October 2022.

In February 2023, she began a 15-day course of nirmatrelvir/ritonavir outside of the context of an acute COVID-19 infection. Concomitant medications included low dose naltrexone, cetirizine, norethindrone acetate-ethinyl estradiol-iron, lumbrokinase, serrapeptase, and palmitoylethanolamide. During her nirmalterlvir/ritonavir course, she reported improvement in her energy and feeling stronger. Her blood pressure was consistently higher during this period (~145/80) and she did not experience orthostatic intolerance. However, she also experienced severe constipation and a severe flare of her MCAS symptoms (e.g., rashes, headaches, increased food intolerances). Two weeks after completing the nirmatrelvir/ritonavir course, she reported improved energy, physical strength, and mental clarity, and complete resolution of symptoms of orthostatic intolerance, which have not recurred as of July 2023. Although she had ongoing MCAS symptoms, these also improved with mast-cell stabilizers H1 and H2 blockers.

### Case #3

In May 2022, a previously healthy 25-year-old woman with a medical history notable only for mild seasonal allergies developed COVID-19 symptoms. The infection was confirmed via rapid antigen test. She had previously received the primary two-shot series of the COVID-19 vaccine (Comirnaty) in April 2021, and a Comirnaty booster in November 2021. Initially, her Long COVID symptoms were primarily gastrointestinal, including lack of appetite, bloating, weight loss, and new food intolerances. Over the next few months, she developed additional symptoms, including fatigue, PEM, exercise intolerance, brain fog, headaches, noise and light sensitivity, and joint pain. In November and December 2022, she was treated for small intestinal bacterial overgrowth with two courses of rifaximin, which did not lead to sustained improvement; specifically, after the first course in November, her gastrointestinal symptoms improved for two days before returning to pre-rifaximin baseline, and after the second course in December, there was no improvement.

In January 2023, she began a 10-day course of nirmatrelvir/ritonavir outside of the context of an acute COVID-19 infection. At the time, she was taking loratadine and a multivitamin. On day 4, she experienced a severe “crash,” characterized by increased fatigue, headache, brain fog, sound and light sensitivity, and difficulty remaining upright, walking, and balancing. After completing the 10-day course, she continued to experience these symptoms for another month. Her fatigue and PEM lessened significantly and she experienced limited improvement in brain fog and headaches. In late February and March 2023, she switched from loratadine to fexofenadine and famotidine. In April and May 2023, her symptoms returned to her pre-nirmaltrevir/ritonavir baseline, and she began taking quercetin, low-dose naltrexone, and a probiotic. As of July 2023, she was experiencing some improvements in symptoms, was slowly reintroducing foods not tolerated earlier in her disease course, and added gentle swimming multiple times per week without PEM. She also began a course of valacyclovir with celecoxib.

### Case #4

In August 2022, a 51-year-old man with a medical history notable only for tinnitus tested positive on PCR for SARS-CoV-2. He had previously received four doses of SARS-CoV-2 vaccines (Comirnaty in April and May 2021, Spikevax in November 2021 and August 2022), most proximally three weeks prior to COVID-19 symptom onset. His acute COVID symptoms included mild cough, fatigue, severe brain fog, heart rate variability, symptoms of paresthesia (skin crawling sensations and tingling in the back), visual snow and mild warping visual distortions, and loss of smell and taste. He began a 5-day course of nirmatrelvir/ritonavir within 24 hours of testing positive, during which he experienced improvement in some symptoms. After completing the 5-day course, he tested negative on day five, then experienced rebound symptoms, testing positive again 2–3 days after his last nirmatrelvir/ritonavir dose. This was associated with worsening cough, fatigue, headaches, brain fog, symptoms of dysautonomia, neuropathy, and inflammatory symptoms including joint pain and range of motion restriction. He tested every 2–3 days, continuing to test positive up until day 18. On day 19, he tested negative, although he continued to experience ongoing cough, brain fog, and exhaustion. He tested negative again on days 20, 22, and 24, then stopped testing regularly. His Long COVID symptoms included fatigue, daily headaches, brain fog, mental exhaustion, PEM, racing heart, palpitations, tinnitus, visual and sensory disturbances, sensitivity to noise, difficulty regulating his emotions, and paresthesia. He began taking fexofenadine, Alpha-Lipoic acid, Acetyl-L-carnitine, which improved energy and inflammation some, and ibuprofen as needed for joint pain, which also seemed to occasionally help brain fog. These symptoms ebbed and flowed but got progressively better, until January 2023, when he experienced a severe crash after trying to exercise, from which he did not recover.

In March 2023, he started a 15-day course of nirmatrelvir/ritonavir outside of the context of an acute COVID-19 infection. He concurrently began taking valacyclovir to address reactivation of Epstein-Barr virus (EBV Early Antigen D AB (IGG) levels were 143) and received a Glutathione/Vitamin-B complex shot two days before. He had never previously had EBV reactivation and was unaware of his original infection date, though antibody levels showed this was not his initial EBV infection. By the end of his first week taking valacyclovir and nirmatrelvir/ritonavir, his energy improved and he felt that he was back to his baseline health for several days. His PEM decreased in frequency, intensity, and duration, requiring him to rest for only 6–36 hours to recover, instead of his usual two weeks. His symptoms of paresthesia resolved and he noted fewer headaches and improvement in his tinnitus, cognition, and affect. He continued to feel symptom reduction for four weeks after the completion of the nirmatrelvir/ritonavir and two weeks after the completion of the valacyclovir courses. After that point, he began to experience “crashes” again with more intensity. He then resumed valacyclovir, experiencing improvement within 24 hours.

As of early June 2023, he reported feeling “radically better” and had returned to 90–95% of his previous health baseline five days per week and 75–85% the other two days. He had not yet returned to aerobic workouts, but was able to walk approximately five miles with a 30-pound backpack and do light weightlifting. He reported substantial improvement in his brain fog, mental exhaustion, affect, sensitivity to noise, symptoms of neuropathy, and ability to exert himself physically and complete resolution of his headaches, problems with regulating his emotions, visual disturbances, palpitations, and tachycardia. In mid-June 2023, he added low-dose naltrexone; since then, he has felt “much more stable” and has experienced no PEM. As of mid July 2023, he reported feeling back to pre-COVID baseline on all symptoms, other than symptoms he attributed to deconditioning. He is slowly reintroducing aerobic exercise.

### Case #5

In March 2020, an adult woman (age not disclosed) with a medical history of recurrent sinus infections developed symptoms consistent with COVID-19. At the time, COVID tests were not available in her location, but she tested negative for flu and strep throat and a clinical diagnosis of COVID-19 infection was made. Her acute symptoms included a high fever, trouble breathing, and cognitive difficulties (e.g., not remembering how to use a phone to call for help). Her primary Long COVID symptoms included respiratory issues, fatigue, PEM, brain fog, and body aches. She had previously been able to exercise daily, but as a result of Long COVID was no longer able to. Laboratory testing was notable for a high anti-nuclear antibody titre as well as the presence of EBV and Guillain-Barré Syndrome (GBS) antibodies, none of which had previously been documented in her medical records (she had previously tested normal for anti-nuclear antibodies and negative for EBV; she had not previously been tested for GBS antibodies). She subsequently received five COVID-19 vaccines (Spikevax), beginning in 2021, at the recommended time intervals for primary series and boosters. Each of these vaccines led to some improvement in her symptoms. She also experienced improved respiratory symptoms after a pneumoccocal-23 vaccination. The only medication she takes is galcanezumab-gnlm for migraines.

In June 2023, she began a 10-day course of nirmatrelvir/ritonavir outside of the context of an acute COVID-19 infection. Within a few days, her severe brain fog disappeared and her respiratory sensitivity and shortness of breath improved. She also saw significant improvement in multiple health metrics, including a lowering in respiratory rate, an improvement in oxygen rate, and a lowering in heart rate (as measured with a wearable device), including during physical exertion activities. She described the cardiovascular improvement after nirmatrelvir/ritonavir as “vast”. For example, she compared her heart rate while doing cardiovascular exercise from three time points: prior to onset of her Long COVID symptoms, during Long COVID pre-nirmatrelvir/ritonavir, and during Long COVID post-nirmatrelvir/ritonavir, and her heart rate did not go up as much post-nirmatrelvir/ritonavir as pre-nirmatrelvir/ritonavir. She was also able to go on several work-related trips without crashing or exacerbating symptoms, which she had not been able to do since her Long COVID began.

### Case #6

In March 2020, a 40-year-old man who was a prior competitive soccer player and mountain climber developed COVID-19 symptoms, including anosmia and shortness of breath. The infection was confirmed with an antibody test in April 2020. His respiratory and inflammatory symptoms persisted for ~10 days, then substantially resolved. Approximately eight weeks post-acute infection, in late May 2020, he began to experience plantar-palmar sensory neuropathies that gradually progressed in intensity and pain/paresthesias that were felt progressively more centrally, as well as a very high frequency of gastrointestinal symptoms, including frequent diarrhea and progressive food sensitivities. In September 2020, he began to develop blurry vision. He received the primary series of the COVID-19 vaccine in March and April 2021 (Comirnaty). After the first shot, he had a severe reaction (grapefruit-sized painful injection site reaction and flared symptoms) for five days, followed by 7 days of total symptom remission (clearer vision, neuropathic symptomology disappeared, return of energy), then a decline for three weeks. After the second shot, he experienced mild tinnitus for a day, but had no reaction at the injection site and no impact on symptoms.

He completed a 5-day course of nirmatrelvir/ritonavir outside of the context of an acute COVID-19 infection in the first quarter of 2022, leading to near-complete remission in symptoms by day 3, with a slow but progressive return of symptoms on day 6. He subsequently took a 30-day course of nirmatrelvir/ritonavir in September 2022, again outside of the context of an acute COVID-19 infection. During this course, he experienced slow remission of symptoms over the first 10 days, with progressive improvement in energy, vision clarity, and cognition over the subsequent weeks. Symptom improvement persisted for nearly two months, with a return of mild symptoms in February 2023. For example, he had had severe gastroesophageal reflux disease that developed as part of Long COVID, which disappeared upon taking nirmatrelvir/ritonavir, but has since returned. He was reinfected, as confirmed by rapid antigen test, during international travel in March 2023, and received a 10-day course of nirmatrelvir/ritonavir, which led to a rapid resolution of acute symptoms. Subsequent detailed serologic analysis of antibodies (Serimmune) in early April 2023 showed an absence of COVID-related antibodies, including those typically induced via vaccination. He was subsequently approved for intravenous immunoglobulin (IVIG), based, in part, on the presence of anti-trisulfated heparin disaccharide antibodies confirmed by multiple labs, and has had a progressive reduction in symptoms over the subsequent six months.

As of July 2023, his symptoms have improved somewhat, including less frequent waking from sleeping due to neuropathy, some brain fog, more mild POTS symptoms, sporadic joint pain in hands, intermittent pain in one of his legs, milder headaches, and being able to add some foods back to his diet (which remains restricted by gastrointestinal symptoms, including diarrhea).

### Case #7

A 29-year-old man had clinically confirmed SARS-CoV-2 infections in May 2022 and July 2022. His symptoms appeared to resolve fully after his first infection. After his second infection, his acute symptoms resolved after three weeks, at which time his Long COVID symptoms arose. His Long COVID symptoms include extreme fatigue, PEM, tachycardia, POTS, persistent headache, brain fog, chest pain and shortness of breath on exertion, and unrefreshing sleep; he met the myalgic encephalomyelitis/chronic fatigue syndrome (ME/CFS) diagnostic criteria.^19^ Since the onset of his Long COVID symptoms, he also developed constipation, chilblains, atopic dermatitis on his face, and paresthesia. His abnormal test results include high cholesterol and prominent Virchow spaces on a brain MRI. Before his initial infection, he had received three doses of the Comirnaty vaccine (March 2021, April 2021, and November 2021). He received an additional dose of the Spikevax vaccine in October 2022.

In May 2023, he began a 15-day course of nirmatrelvir/ritonavir outside of the context of an acute COVID-19 infection. Concomitant medications included finasteride, amphetamine/dextroamphetamine salts, bupropion, atenolol, midodrine, Vitamin D3, and famotidine. Approximately six days into the course, he noticed slight (~10%) improvement in his fatigue and brain fog. These improvements were sustained and increased to about 20% by the end of the course. His 15 days on nirmatrelvir/ritonavir also coincided with the longest number of consecutive days (15) without any worsening of symptoms (e.g., a crash). However, two days after the final nirmatrelvir/ritonavir dose, he experienced a PEM crash, which included a sore burning sensation in his arms, poor sleep, headache, fatigue, and brain fog so intense that sitting, moving, and talking was difficult. He returned to his pre-nirmatrelvir/ritonavir Long COVID baseline within five days. He has remained at his pre-nirmatrelvir/ritonavir Long COVID baseline in the subsequent months.

### Case #8

In July 2020, a 35-year-old woman who was a prior long-distance runner developed a clinically confirmed SARS-CoV-2 infection. While she was not hospitalized during her acute COVID infection, she experienced loss of taste and smell, fever, sore throat, fatigue, and substantial difficulty breathing. After the acute phase, she continued to experience symptoms consistent with Long COVID, including fatigue, PEM, and migraines, with her migraines manifesting as the most debilitating aspect of her PEM. Her difficulty breathing slowly resolved over six months; she experienced chest pain starting two months after the acute infection and largely resolving over about two years. As part of her Long COVID symptoms, she was diagnosed with mild ME/CFS in January 2021, which has since progressed to moderate ME/CFS. Since her infection, she has received five COVID vaccinations (Comirnaty primary series in April and May 2021, Spikevax booster in November 2021, Comirnaty boosters in June 2022 and October 2022).

In April 2023, she initiated an extended course of nirmatrelvir/ritonavir outside of the context of an acute COVID-19 infection. Concomitant medications included acetaminophen, sumatriptan, Excedrin, ubuiquinone, taxifolin, a multivitamin, Vitamins C, D, and E, and zinc. For the first ten days, she took half doses of nirmatrelvir/ritonavir (spreading a five-day course over ten days). For the subsequent five days, she took full doses of nirmatrelvir/ritonavir, completing an additional five-day course. During this time, she experienced some insomnia and taste-related side effects, but did not observe any change in her Long COVID symptoms.

### Case #9

In November 2021, a 34-year-old-woman developed symptoms consistent with a COVID-19 infection, confirmed by rapid antigen test. She had previously received two doses of the Comirnaty SARS-CoV-2 vaccine in October and November 2021. Following the acute illness, she felt herself to have recovered, with no lingering symptoms other than a feeling of being “wired.” Approximately one week after the acute infection, she went for a run and experienced a first episode of PEM 24 hours later that caused her to collapse, and she was hospitalized as a result. Extensive laboratory testing was notable only for mild anemia. This episode of PEM precipitated three months of symptoms consistent with severe ME/CFS in which she was largely bedbound and dependent on others for care. During this time, she experienced fatigue, brain fog, short-term memory loss, and difficulty finding and remembering words. Her symptoms also met the diagnostic criteria for dysautonomia, POTS, nerve damage, and small fiber neuropathy. Self-treatment with cannabinoid resulted in some improvement, which shifted her from severe to moderate ME/CFS (mostly housebound, by ICC criteria). ^20^

In May 2022, she took a 15-day course of nirmatrelvir/ritonavir outside of the context of an acute COVID-19 infection. Her only concomitant medication was aspirin. She did not experience any change in her symptoms during or after the nirmatrelvir/ritonavir course. Since that time, her doctor has treated her ME/CFS with rapamycin, which has led to substantial relief in symptoms.

### Case #10

In January 2022, a 45-year-old woman developed symptoms of a COVID-19 infection. Her Long COVID symptoms included fatigue, breathing difficulties, and severe chest pain diagnosed as costochondritis. Over the first eight weeks of her illness, her Long COVID symptoms worsened and she experienced severe weight loss and migraines. Since that time, she has been diagnosed with chronic pericarditis, POTS, cardiac fibrosis, ME/CFS, and possible MCAS. Before her illness, she had received two doses of the Comirnaty vaccine (May and July 2021) and a dose of the Spikevax vaccine in January 2022 (immediately prior to her diagnosis). She received an additional dose of the Spikevax vaccine in September 2022.

In October 2022, she completed a 5-day course of nirmatrelvir/ritonavir in the setting of a SARS-CoV-2 reinfection. She experienced three days with no fatigue and in which she described “feeling normal.” However, these improvements did not persist, and she returned to her pre-reinfection baseline. Because of this temporary improvement, she completed a second, 15-day course of nirmatrelvir/ritonavir in late November 2022 outside of the context of an acute COVID-19 infection. This additional course of nirmatrelvir/ritonavir did not improve her fatigue or her other symptoms in any way.

### Case #11

A 33-year-old woman developed symptoms of a SARS-CoV-2 infection in March 2020 (clinically confirmed via emergency room telehealth). For the first two weeks of her illness, her symptoms included sore throat and excessive daytime fatigue and sleepiness. Shortly thereafter, her condition worsened considerably and she felt severely ill for another 3–4 weeks. This left her bedridden, only able to go to the bathroom on her own (which required resting before crawling back to bed), and a similar crawling and resting routine to get any food 1–2 times daily. Her symptoms included extreme fatigue and PEM with myalgia, sensory sensitivity and flu-like symptoms lasting days or weeks; POTS with dizziness, temperature irregularity, and low blood pressure; insomnia and unrefreshing sleep; joint and muscle pain exacerbated by PEM; gastrointestinal issues with alternating constipation and diarrhea; no libido; and cognitive issues affecting concentration, word finding, memory, and information processing. While she experienced some symptom improvement over time, other symptoms (e.g., fatigue, confusion, headache, myalgia) increased in severity through spring 2021, and she became bedridden again in spring 2022. Since her initial infection, she received the Jcovden vaccine in April 2021, a dose of the Comirnaty vaccine in November 2021, and a dose of the Nuvaxovid vaccine in November 2022.

In May 2022, she took a 5-day course of nirmatrelvir/ritonavir outside of the context of an acute COVID-19 infection, experiencing no side effects other than dysgeusia. This course led to improvements in overall functioning, such that she was no longer bedridden and other symptoms were less severe (e.g., fatigue improved, migraine improved, light and sound sensitivity reduced enough to be able to leave dark bedroom for short periods of time, shifted from needing to be fed while lying down to being able to sit up in bed to eat and drink). Concomitant medications included escitalopram, propranolol, low-dose naltrexone, pravastatin, fenofibrate, and maraviroc. In June 2022, she attempted a second 5-day course of nirmatrelvir/ritonavir, also outside of the context of an acute COVID-19 infection. She experienced severe stomach pain within 5 hours of taking the first dose and discontinued the course. This stomach pain lasted 7–9 hours and she had gastrointestinal issues for the next week. At the time of the second course, concomitant medications included those listed above, as well as clopidogrel and meloxicam. Suspecting that the stomach pain was due to contraindicated medications, she attempted a third, 15-day course of nirmatrelvir/ritonavir in February 2023, also outside of the context of an acute COVID-19 infection. At this time, she stopped all of her other medications except escitalopram, propranolol, and low-dose naltrexone. She again experienced severe stomach pain within 5 hours of the first dose of nirmatrelvir/ritonavir and stopped the course. As before, her stomach pain lasted 7–9 hours, with gastrointestinal issues lingering for the next week.

The improvements from the initial 5-day course of nirmatrelvir/ritonavir have persisted, but she continues to experience her same Long COVID symptoms and significant functional limitations.

### Case #12

In December 2020, a 41-year-old physically fit woman who ran marathons developed symptoms of a COVID-19 infection, confirmed by a positive PCR test. During the acute phase of her COVID infection, she experienced body aches, dizziness, vertigo, gastrointestinal issues, difficulty breathing, and chest pain. After the acute phase, her dizziness, vertigo, chest pain, and difficulty breathing continued and she developed widespread tingling and numbness, sensitivity to bright lights and loud noises, and PEM. She was unable to return to work for nine months. Since her initial infection, she has had four shots of the Spikevax COVID-19 vaccine: her primary series in March/April 2021, and boosters in January 2022 and November 2022.

She experienced slow improvements in her symptoms starting six months after initial infection. Her vertigo, difficulty breathing, gastrointestinal issues, and nose bleeds improved in the summer of 2021. She used pacing as a strategy to avoid PEM, and returned to work in September 2021. Throughout 2022, her symptoms slowly improved and she found daily stretches helped with the chest pain. By February 2023, her only remaining Long COVID symptom was chest pain, managed by the daily stretching. She had returned to running and lifting weights again.

In April 2023, she was reinfected with SARS-CoV-2 and was prescribed a 10-day course of nirmatrelvir/ritonavir. Concomitant medications included zinc, vitamin C, vitamin D, astragalus, echinacea, and melatonin. She had also taken one dose of ivermectin three days prior to starting nirmatrelvir/ritonavir. On the seventh day (after 13 doses) of nirmatrelvir/ritonavir, she experienced itchiness and tingling throughout her body (legs, abdomen, neck) and in her mouth and throat. She discontinued the course and her itchiness resolved by the next morning. For approximately the next two weeks, she experienced dizziness, and shakiness and tingling in her arms and hands. She took Tailwind Endurance Fuel to ease these symptoms. Two weeks after stopping the nirmatrelvir/ritonavir, she had returned to her previous baseline. However, within another two days, she developed a new sore throat and experienced tingling and dizziness. She then tested positive again for SARS-CoV-2 on a rapid antigen test. She completed the remaining three and a half days (seven doses) of nirmatrelvir/ritonavir, noting improvement in her symptoms. One week after finishing nirmatrelvir/ritonavir, she continued to experience tingling and dizziness and brain fog and had not returned to her previous baseline. Two months later, she had returned to her pre-April 2023 reinfection baseline.

### Case #13

A 55-year-old transgender person assigned female at birth developed symptoms of a clinically confirmed COVID-19 infection in March 2020 and a reinfection in March 2021 (confirmed by PCR test). Before his illness, he had been diagnosed with Neuromyelitis Optica Spectrum Disorder (2011), ME/CFS (diagnosed in 2017, but began earlier), fibromyalgia, and psoriasis. Following his first COVID-19 infection, he developed tachycardia, brain fog, and mild cognitive symptoms. After his second COVID-19 infection, he developed MCAS symptoms, including frequent, mild-to-moderate sinus congestion, flushing, and throat swelling in relation to many foods. Currently, his Long COVID symptoms include brain fog, difficulty with word finding, MCAS symptoms, sensitivity to histamines, and intermittent PEM. He received one dose of the Spikevax vaccine in February 2021, two doses of the Jcovden vaccine in July and October 2021, a Comirnaty bivalent booster in October 2022, and one primary series dose of the Nuvaxovid vaccine in April 2023.

In December 2022, he took a 7.5 day course of nirmatrelvir/ritonavir during a third COVID-19 infection (confirmed by at-home molecular Lucira test and multiple rapid antigen tests). At the time, he was taking cromolyn sodium, ketitofen, low-dose naltrexone, metoprolol succinate XL, rosuvastatin, nortriptyline, modafinil, finasteride, loratadine, valacyclovir, famotidine, and nitrofurantoin daily; testosterone cypionate weekly; leuprolide quarterly; and Zofran and clonazepam as needed. His daily supplements included Deplin (algal oil), cranberry extract, laxin forte, vision optimizer blend, biotin, ubiquinol, alpha-lipoic acid, probiotics, fish oil, flaxseed oil, quercetin, vitamin C, acetyl-l-carnitine, milk thistle, P5P, calcium, skullcap and passionflower tinctures, and DAO and digestive enzymes. He also received twice-weekly injections of glutathione and methylcobalamin. During his acute COVID infection, he also took melatonin and high dose Vitamin C powder.

After this course of nirmatrelvir/ritonavir, he experienced several days with no fatigue or significant pain, although his fatigue and pain then returned to his pre-infection baseline within 2–3 weeks. This pattern resembled his experience with his first COVID infection, in which he experienced improvements in his fatigue and chronic pain during the ten days of initial infection, while also experiencing intermittent low-grade fevers. At that time, as his fevers subsided, his fatigue and pain returned.

## Discussion

This case series describes 13 individuals with established Long COVID who reported variable outcomes following extended antiviral treatment with nirmatrelvir/ritonavir (summarized in Table 1). Although the treatment was generally well-tolerated, these individuals exhibited heterogeneity in the clinical manifestations of Long COVID and the impact of antiviral therapy on their symptoms. Given the lack of accepted treatments for Long COVID, these cases contribute to burgeoning research suggesting that at least some individuals may benefit from antiviral therapy beyond the acute phase of infection. Taken together, this case series provides a strong rationale for the study of extended courses of nirmatrelvir/ritonavir and/or other antiviral medications in randomized clinical trials, some of which are underway, with the goal of informing clinical recommendations for using antivirals as a potential treatment option for Long COVID.

There is currently debate as to whether a 5-day duration of nirvmatrelvir/ritonavir therapy is sufficient in all cases during acute SARS-CoV-2 infection. In addition, some individuals initially reporting improvement in Long COVID symptoms have described a recurrence of symptoms following the 5-day course. For this reason, there is growing interest in the safety, tolerability, and efficacy of longer courses of antivirals like nirmatrelvir/ritonavir. Although the mechanism of persistence is different, there is precedent for longer courses of antivirals in the protease inhibitor class in both HIV and hepatitis C. To the best of our knowledge, this is the first case series of people with Long COVID who have attempted extended courses of nirmatrelvir/ritonavir, with most reporting their experiences outside of the context of an acute COVID-19 infection.

There are several considerations for future research assessing antivirals in established Long COVID. First, there is growing consensus that there are likely to be different endotypes of Long COVID, which may be driven by different underlying biological processes. Ignoring this heterogeneity may make it difficult to identify a signal, if persistent viral replication drives only some cases of Long COVID. For this reason, clinical trials should further explore and attempt to phenotype individuals who exhibit a benefit, even if this signal is lost in the larger group of participants. In larger studies, we recommend enrolling adequate numbers of different phenotypes (by symptom clusters for now, until more specific phenotypes are identified), as is being attempted in at least one study of nirmatrelvir/ritonavir (NCT05595369). Second, prolonged courses of these agents may be worth further study. In this case series, some individuals experienced improvement only after ≥10 days of treatment. It will be important to trial a variety of lengths of time in order to determine if sustained resolution of symptoms is possible with longer courses. Third, that some individuals experienced recurrence of previously improved symptoms following discontinuation of antiviral treatment suggests that researchers and regulators need to consider the potential impact of treatment completion on participants and determine whether mechanisms to allow for ongoing treatment can be implemented to limit this symptomatic rebound. Fourth, although treatment appeared to overall be safe in these individuals, some participants experienced flares of MCAS-related symptoms and/or post-exertional malaise. Such symptoms must be monitored closely to determine whether they are specifically related to antiviral therapy, and if so, this risk must be considered in addition to the known potential risks related to drug-drug interactions and other adverse effects.

If clinical trials do indeed detect a signal, there should be a clear regulatory pathway to ensure that these drugs can be made accessible to patients, especially since there are currently no FDA-approved treatments specifically for Long COVID. This might entail consideration of options like expanded access, fast-track designation, and breakthrough therapy designation to both encourage industry participation and increase access of pharmaceutical treatments to Long COVID patients.

A major strength of this study is its grounding in patient-led and participatory approaches to draw from knowledge outside of traditional medical institutions. While we were unable to conduct independent reviews of medical records, the patients’ own descriptions of their experiences provide insights that may otherwise not be captured due to limitations in medical record systems, including inaccuracies in recording symptoms and variability in healthcare system contact.^21^ This is particularly the case with emerging illnesses like Long COVID that did not have its own ICD-10 diagnostic code until October 2021,^20^ 1.5 years after some of the participants in this case series began experiencing Long COVID. What we report here- the lived experience of people with Long COVID who were able to access this treatment and report on its perceived impact- continues to build upon efforts by people experiencing Long COVID to describe and study the condition, which has been critical to driving forward this field of research. This approach may be useful for studying potential treatments for other infection-associated chronic conditions as well.

In conclusion, we describe the use of long courses of Paxlovid as a potentially promising pharmaceutical treatment to support at least some people with Long COVID. These cases provide strong rationale for the ongoing study of antivirals for Long COVID to determine if, when, and how they should be used in this patient population.

## Figures and Tables

**Table 1.** Summary of cases

Case #	Age, gender	Date of COVID infection(s)	Suspected variant	Initiation of Long COVID symptoms	COVID vaccinations	Date and length of extended course of Paxlovid	Short-term improvement with Paxlovid	Sustained improvement with Paxlovid	Adverse effects of Paxlovid
1	56 y.o. man	March 2020	original wild type	July 2020	Feb 2021, Mar 2021, Nov 2021, May 2022, Nov 2022	January 2023 (15 days)	y	y	
2	45 y.o. woman	March 2022	omicron	April 2022	Jan 2021, Feb 2021, Nov 2021, Oct 2022	Feb 2023 (15 days)	y	y	y
3	25 y.o. woman	May 2022	omicron	May 2022	April 2021, April 2021, Nov 2021	January 2023 (10 days)	y		y
4	51 y.o. man	Aug 2022	omicron	August 2022	April 2021, May 2021, Nov 2021, Aug 2022	March 2023 (15 days)	y	y	
5	woman, age undisclosed	March 2020	original wild type	March 2020	5 vaccines beginning in 2021	June 2023 (10 days)	y	y	
6	40 y.o. man	March 2020	original wild type	May 2020	Not specified	Sept 2022 (30 days)	y	y	
7	30 y.o. man	May 2022, July 2022	omicron, omicron	July 2022	Mar 2021, April 2021, Nov 2021	May 2023 (15 days)	y		
8	35 y.o. woman	July 2020	original wild type	July 2020	April 2021, May 2021, Nov 2021, June 2022, Oct 2022	April 2023 (10 days)			
9	34 y.o. woman	Nov 2021	delta	November 2021	Oct 2021, Nov 2021	May 2022 (15 days)			
10	45 y.o. woman	Jan 2022	omicron	January 2022	May 2021, July 2021, Jan 2022, Sept 2022	Nov 2022 (15 days)			
11	37 y.o. woman	March 2020	original wild type	March 2020	April 2021, Nov 2021, Nov 2022	Feb 2023 (15 days attempted)			y
12	41 y.o. woman	Dec 2020	alpha	Dec 2020	Mar 2021, April 2021, Jan 2022, Nov 2022	April 2023 (10 days) (during acute infection)	(returned to prereinfection baseline within 2 months)		y
13	55 y.o. transgender person	Mar 2020, Mar 2021, Dec 2022	original wild type, alpha, omicron	March 2020	Feb 2021, July 2021, Oct 2021, Oct 2022, April 2023	Dec 2022 (7.5 days) (during acute infection)	(returned to prereinfection baseline within 3 weeks)		
